# Turkish validity and reliability of the Self-Report Instrument to Measure Patient Safety Attitudes, Skills, and Knowledge

**DOI:** 10.1186/s12912-024-02091-9

**Published:** 2024-06-26

**Authors:** Nuray Turan, Seçil Erden Melikoğlu, Zühal Nas

**Affiliations:** 1 Department of Fundamentals of Nursing, Faculty of Nursing, Istanbul University, İstanbul, Türkiye; 2 Department of Fundamentals of Nursing, Florence Nightingale Faculty of Nursing, Istanbul University-Cerrahpasa, İstanbul, Türkiye; 3 Department of Ophthalmology, Cerrahpaşa Medical Faculty, Istanbul University-Cerrahpaşa, İstanbul, Türkiye

**Keywords:** Skills and knowledge, Reliability, Validity, Turkish nurses

## Abstract

**Aims:**

The present study was carried out methodologically to provide the Turkish equivalence of the Self-Report Instrument to Measure Patient Safety Attitudes, Skills, and Knowledge and to determine its reliability and validity.

**Methods:**

This methodological study included 317 nurses. The back-translation method was used to test the linguistic equivalence of the methodological scale. Experts’ opinions were asked to test its content validity. Time invariance (test-retest reliability) and internal consistency were tested to test its reliability. A group of 100 nurses participated in the test-retest. The content validity index and confirmatory factor analysis were used to test its validity.

**Results:**

The scale was highly reliable, with a content validity index value of 0.965 and an overall internal consistency coefficient of 0.875. Confirmatory Factor Anaysis (CFA) showed that the goodness-of-fit indices were good and that the model was suitable for this situation.

**Conclusions:**

The Turkish version of this scale is reliable and valid for evaluating nurses’ knowledge and attitudes about patient safety and their perceptions of patient safety culture. Therefore, it is possible to apply this approach in studies carried out in Türkiye.

## Introduction

Patient safety has emerged as a crucial issue in healthcare, particularly as preventable harms to healthy and sick individuals during healthcare practices have become increasingly evident. Ensuring and maintaining individual safety remains a priority for healthcare institutions, as highlighted by numerous studies [1–3]. Patient safety encompasses all measures taken by healthcare institutions and their employees to prevent harm during the delivery of healthcare services [4–6]. A stark report from the Institute of Medicine in the United States points out that the number of deaths due to medical errors surpasses those caused by traffic accidents, breast cancer, or AIDS, marking medical errors as a significant patient safety concern [7, 8]. Similarly, the World Health Organization’s 2004 report underlined the importance of patient safety and discussed necessary strategies to ensure the delivery of healthcare services in a safe and quality manner [9].

The International Council of Nursing (ICN) emphasizes the critical role of employing professional healthcare workers to enhance patient safety. This involves improving performance, combating infections, and ensuring the safe use of drugs and auxiliary equipment [10]. Comprehensive measures are necessary in healthcare settings, including environmental safety and risk management. Combining patient safety-focused scientific knowledge with supportive infrastructure is crucial for fostering patient development. Since the 1990s, with the spread of health reform movements in Turkey, patient safety has become a significant discussion point among healthcare professionals [5–11].

Healthcare professionals encounter many situations that threaten patient safety in healthcare delivery. They have a vital duty to protect healthy and sick individuals and their families from potential dangers and ensure the safe maintenance of healthcare practices. As the largest group among healthcare professionals, nurses constantly contact patients and assume a privileged role in ensuring patient safety [1, 7, 12–14]. Nurses’ legal and ethical responsibilities to maintain and ensure patient safety are inseparable from their nursing care. By identifying potential harms and being aware of the factors affecting patient safety, nurses play a critical role in enhancing the well-being of individuals [15].

In this context, it is essential for nurses to assess their level of knowledge, attitudes, and perceptions of patient safety culture [3, 6, 16, 17]. Despite the critical importance of these components, a scale that evaluates all three aspects of patient safety competencies—knowledge, attitudes, and behaviors—has yet to be developed in our country. The significant contribution of this study is the provision of a scale that determines the knowledge, attitudes, and behaviors of nurses in our country concerning patient safety. This study also aids other healthcare team members in assessing nurses’ knowledge, attitudes, and perceptions of patient safety culture, thereby increasing awareness of this critical issue. This methodological research is specifically aimed at determining the validity and reliability of the Self-Report Instrument for Measuring Patient Safety Attitudes, Skills, and Knowledge, which is internationally recognized for evaluating the knowledge, attitudes, and behaviors related to patient safety [17].

## Materials and methods

### Study design and participants

The present study was carried out methodologically to provide the Turkish equivalence of the Self-Report Instrument to Measure Patient Safety Attitudes, Skills, and Knowledge and to determine its reliability and validity. Nurses working at a university hospital in Istanbul composed the study population between July 2020 and July 2021. Based on the suggestion that the sample size needs to be 5–10 times greater than the number of scale items needed to perform statistical analyses in methodological research, the sample size was calculated to be 317, ten times the number of items for the 26-item scale [18]. Initially, 317 nurses were selected for the study using a simple random sampling method. To determine invariance over time, 100 nurses were reached for the test-retest analysis. The study inclusion criteria were nurses who were not on sick leave at the time of the research and were willing or volunteering to participate. The exclusion criteria for the study were nurses who were on sick leave at the time of the research or those who were not willing or volunteering to participate.

### Measurements

#### Nurse information form

The form developed by the researchers in literature [3, 6, 16, 17] consisted of questions about nurses’ demographic characteristics (age, sex, education level, marital status, income level), professional characteristics (total working time in the profession, working department, working time in the department, working type, duty in the department, weekly working hours, number of patients provided with care in a shift/overtime), and status of receiving education on patient safety.

### Self-report instrument to measure patient safety attitudes, skills, and knowledge

Schnall et al. [19] developed this instrument to evaluate healthcare professionals’ knowledge, attitudes, and behaviors related to patient safety. This scale comprises 26 items and three subscales. The Attitude subscale of the scale consists of three subscales—knowledge, attitude, and behavior—and consists of 9 items; the behavior and knowledge subscales consist of 13 items and four items, respectively. The reverse of the items (items 3, 4, 7, 8, and 9) were used in the score calculation for the 5-point Likert-type scale. The original scale’s Cronbach’s alpha value was 0.71 [19].

### Linguistic equivalence and content validity of the self-report instrument for measuring patient safety attitudes, skills, and knowledge

**Fig. 1:**
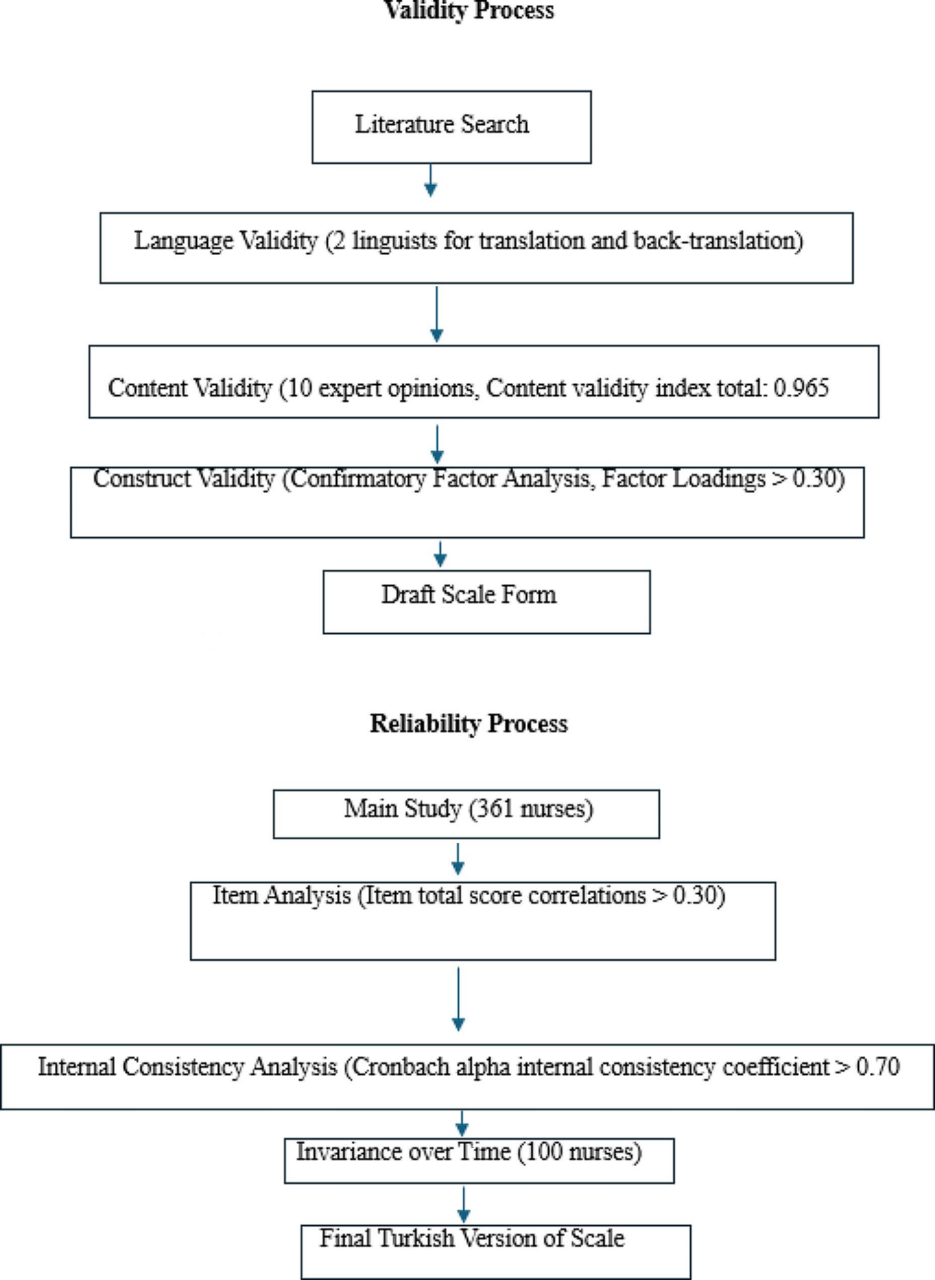
Research process

The scale was independently translated into Turkish by three relevant experts with good English command to ensure the scale’s linguistic equivalence. The scales were subsequently translated into Turkish and examined by an expert lecturer and a Turkish language and literature expert. Conflicting statements were negotiated and corrected, and the scales were made into a single form. The Turkish scale was given to two people with a good command of English, the relevant culture, and Turkish, and they were requested to translate the scale into English. The Self-Report Instrument to Measure Patient Safety Attitudes, Skills, and Knowledge, translated back into English, was compared with the original scale, and the consistency of logic and statements was determined (Fig. 1).

Content validity is essential, especially in scale development studies [11]. The “content validity index” was used to test the scale’s content validity, which was adapted into Turkish by this method. The validity test aims to create a whole consisting of meaningful items by examining whether the domain/behavior to be measured is represented by the items in the measurement tool by a group of experts. Methods to ensure content validity include expert opinion, content analysis, and revision processes. Experts evaluated the Turkish form obtained after translation regarding language and content validity. Confirmatory Factor Analysis (CFA) was performed for the item-factor structure in the original scale to test the model fit. The intelligibility of each item on the scale and whether the items were adequately, accurately, clearly, or unambiguously expressed were evaluated in the range of 1–4 points (1 = not suitable, 2 = slightly suitable, 3 = quite suitable, and 4 = extremely suitable) according to the Davis technique. At least 80% of the experts were expected to rate the scale items as quite or very suitable (Fig. 1).

As a result of the data collection, the Self-Report Instrument to Measure Patient Safety Attitudes, Skills, and Knowledge was used to measure patient safety attitudes, skills, and knowledge; this instrument was subsequently used in statistical reliability and validity studies.

### Reliability and validity of the self-report instrument for measuring patient safety attitudes, skills, and knowledge

The test-retest and internal consistency methods were used to test the scale’s reliability, and the content validity index. and confirmatory factor analysis were used to test its validity. For test-retest reliability, an interval of at least two weeks is usually chosen to minimize memory recall without allowing significant changes in the measured variable [18]. Confirmatory Factor Analysis (CFA) was used to evaluate the suitability of the adapted scale’s original structure. Before performing CFA, the Kaiser Meyer Olkin (KMO) sample adequacy test and Bartlett’s sphericity test were performed to determine sample adequacy (Fig. 1).

### Data collection procedure

Data were collected by the researchers, who distributed them to all nurses after explaining the purpose, content, scope, and what is expected from students who accepted to become participants. The data obtained in the scope of test–repeat are included in the study data. In the test-retest, the students participating in the study were asked to write their nicknames. The researchers readministered the scale for the same student group after a 2-week interval to determine the scale’s time invariance and preferred to apply the same nicknames. The researcher distributed the questionnaires to the nurses in-person during breaks during working hours in a way that did not affect the patient’s care and treatment hours. Then, the researcher collected the forms filled out by the nurses in-person. The average time to complete the questionnaires was 20 min.

### Data analysis

IBM SPSS Statistics 22 and SPSS AMOS 22 (IBM SPSS, Turkey) were used to perform the statistical analysis of the results. Kolmogorov‒Smirnov tests, Q‒Q tests, and histograms were used to evaluate the variables’ compliance with a normal distribution. In addition to descriptive statistical methods (mean, standard deviation, frequency, percentage) used to evaluate the study’s data, Student’s t-test was used to evaluate the differences in quantitative data between two groups, and one-way analysis of variance (ANOVA) was used to evaluate more than two groups. Levene’s test was employed to test the assumption of homogeneity of variances. In determining the groups that caused the difference due to the ANOVA test, the Tukey HSD post hoc test evaluated those with homogeneous variances. Confirmatory Factor Analysis (CFA) was employed for the validity of the scale, the content validity index was employed to evaluate content validity, Cronbach’s alpha coefficient was used in internal consistency analysis in the reliability analysis, Pearson correlation analysis was employed for item-total score correlation, and the intraclass correlation coefficient was employed in the analysis of test-retest reliability. To determine test-retest reliability, Intraclass Correlation (ICC) was administered, and the model fit used GFI, CFI, NFI, RFI, and IFI, and *p* < 0.05 was considered to indicate statistical significance.

### Ethical considerations

Ethical committee approval was received to conduct the study from the Clinical Research Ethics Committee of the university hospital where the data were collected (59491012-604.01.02). First, the authors who developed the scale were informed in writing to use the scale, and approval was received for adapting the scale to Turkish. The researchers explained the aim and benefits of the study and the roles of the nurses who composed the sample, and verbal and written consent was obtained. In the present study, the data were collected from nurses during specific periods (resting time, etc.) that would not affect the care and treatment of patients.

## Results

### Sociodemographic characteristics of nurses

Of the nurses included in the study, 85.2% were female, the mean age was 31.34 ± 8.20 (min.-max: 20–60) years, 57.7% were married, 59.9% had a bachelor’s degree, and 52.7% had less income than expenses. It was determined that while 93.4% of the nurses were clinical nurses, 34.7% worked in surgical departments, 77% worked in shifts, 55.5% provided care for 1–10 patients, and 87.7% had an average weekly working hours of 40 h or less. When the average working hours of the nurses in the profession and their departments and their weekly average working hours were examined, they were found to be 9.10 ± 8.17 (min–max.: 0.5–33) years, 5.66 ± 6.41 (min.-max:0.008-33) years and 40.85 ± 2.71 (min.-max: 25–56) hours, respectively (Table 1).

**Table 1:** General characteristics of nurses (*N* = 317)

General Characteristics of Nurses		Min-Max	Mean ± SD (Median)
**Age (years)**		20–60	31.34 ± 8.20 (30)
**Working time (years)**		0.50–33	9.10 ± 8.17 (7)
**Duration of employment in** **the unit (years)**		0.08-33	5.66 ± 6.41 (2.5)
**Working hours per week**		25–56	40.85 ± 2.71 (40)
		**n**	**%**
**Age group**	**< 30 years**	157	49.5
	**≥ 30 years**	160	50.5
**Gender**	**Woman**	270	85.2
	**Male**	47	14.8
**Marital status**	**Married**	134	42.3
	**Single**	183	57.7
	**Health vocational high school**	60	18.9
**Education status**	**Associate degree**	20	6.3
	**License**	190	59.9
	**MA/PhD**	47	14.8
**Working time**	**< 7 years**	156	49.2
	**≥ 7 years**	161	50.8
**Income status**	**Income covers expenses**	150	47.3
	**Income does not cover expenses**	167	52.7
	**Internal units**	74	23.3
	**Surgical units**	110	34.7
**Unit worked in**	**Gynecology service/ delivery room**	17	5.4
	**Emergency service**	22	6.9
	**Intensive care unit**	73	23
	**Pediatric units**	21	6.6
**Duration of work in the** **unit**	**< 3 years**	161	50.8
	**≥ 3 years**	156	49.2
	**Matron**	2	0.6
**Position in the unit**	**Nurse in charge**	19	6.0
	**Ward nurse**	296	93.4
	**Continuous daytime**	64	20.2
**Way of working in the unit**	**Constantly at night**	9	2.8
	**Shifts**	244	77
**Weekly average** **working hours**	**≤ 40 h**	278	87.7
	**> 40 h**	39	12.3
**Average number of patients cared for**	**1–10 patients**	176	55.5
	**11–19 patients**	112	35.3
	**20 or more patients**	29	9.1
**Participation in training on** **patient safety**	**Yes**	240	75.7
	**No**	77	24.3

(*) Outpatient clinic. training nurse. baby care room. IVF

### Validity of the scale

#### Content validity

The scale is restructured based on the experts’ suggestions and critiques. Content validity is defined as the degree to which the measurement tool as a whole and each item in the measurement tool serve the aim of the test [20].

Ten experts evaluated the language and content validity of the Turkish version obtained after translation. The intelligibility of each item on the scale and whether the items were adequately, accurately, clearly, or unambiguously expressed were evaluated in the range of 1–4 points (1 = not suitable, 2 = slightly suitable, 3 = quite suitable, and 4 = extremely suitable) according to the Davis technique [21]. The scale was expected to be rated as quite or very suitable by at least 80% of the experts [22, 23]. During the evaluation of the items, each item’s Content Valdity Index (CVI) was obtained by dividing the number of experts who chose option (3) or (4) by the total number of experts. The CVI values ​​achieved in the study ranged between 0.800 and 1.000. The CVI was “perfect” for the overall scale, with a value of 0.965. Therefore, it was found that the CVI values ​​were more significant than the generally accepted value of 0.800, and it was determined that the scale items were suitable for language and content validity.

### Construct validity (factor analysis)

Confirmatory Factor Analysis (CFA) was performed to determine the scale’s construct validity. CFA is one of the two most commonly used methods for examining construct validity in scale adaptation studies. Factor analysis aims to express many items with a smaller number of factors. The items measuring the same factor come together to form various groups, and each factor group is given a name according to the features of the items in it [24, 25].

### Confirmatory factor analysis (CFA)

We aimed to determine whether the factor structure of the original form of the scale could be validated in a Turkish sample. The confirmatory factor analysis revealed the model’s competence tested for compatibility using several goodness-of-fit indices. The chi-square fit test, normalized chi-square test (NC), goodness-of-fit index (GFI), root mean square error of approximation (RMSEA), comparative fit index (CFI), normalized fit index (NFI), relative fit index (RFI), and increased fit index (IFI) were analyzed as goodness-of-fit indices for the confirmatory factor analysis performed in this study. An NC value of 2.5 or less indicates a perfect fit. When the fit was considered 0.90, the ideal fit was considered 0.95 for the GFI, CFI, NFI, RFI, and IFI. An acceptable fit value of 0.08 and a perfect fit value of 0.05 were considered for the RMSEA [25]. The goodness-of-fit indices obtained for the confirmatory factor analysis in this study are presented in Table 2.

**Table 2:** Goodness-of-fit indices obtained for the confirmatory factor analysis

Indices	Pre-Modification	Post-Modification
	(χ^2^ = 1196.114/sd = 296)**	(χ^2^ = 696.111/sd = 284)**
**NC (normed chi-square)**	4.041	2.451
**GFI (goodness of fit index)**	0.755	0.852
**RMSEA (root mean square error of approximation)**	0.098	0.068
**CFI (comparative fit index)**	0.757	0.889
**NFI (normed fit index)**	0.703	0.827
**RFI (relative fit index)**	0.673	0.802
**IFI (incremental fit index)**	0.758	0.890

*χ*^*2*^: *chi-square fit test df: degree of freedom* **p < 0.01

**Fig. 2:**
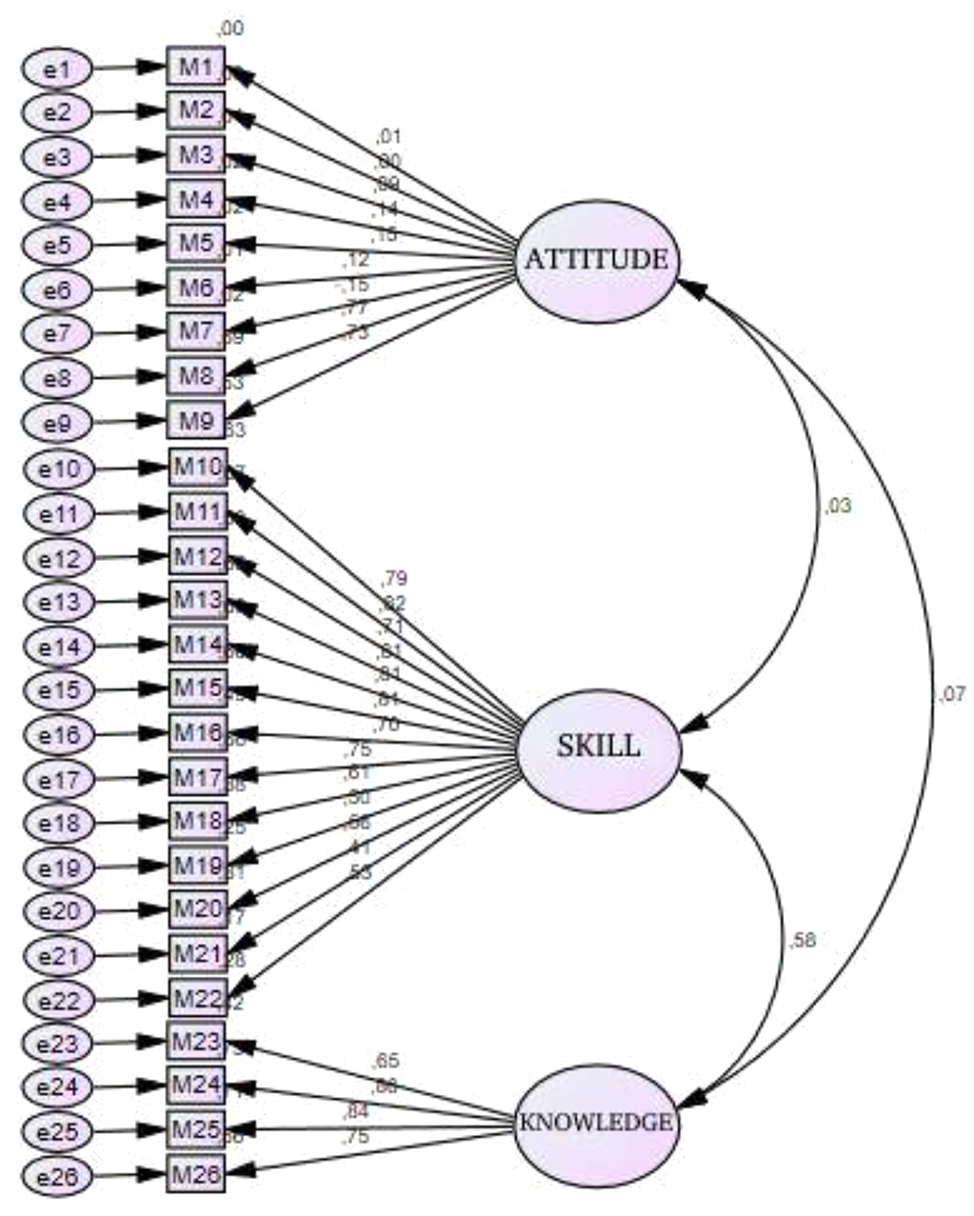
Premodification path diagram and factor loadings (Standardized Estimates)

**Fig. 3:**
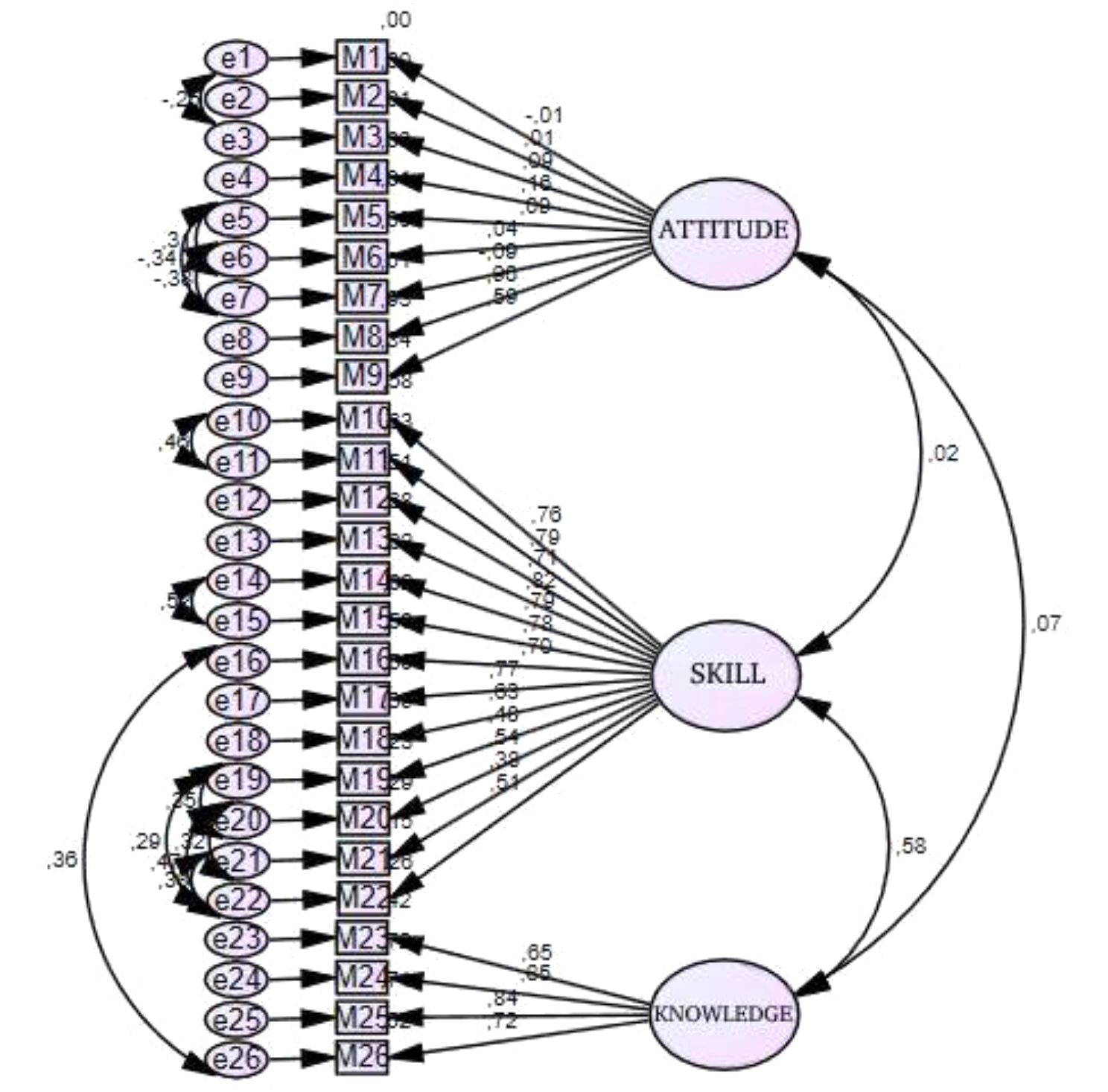
Postmodification path diagram and factor loadings (Standardized Estimates)

In the confirmatory factor analysis, the goodness-of-fit indices of the three-factor model of the Turkish form were examined. In the CFA, modifications were made between items 1 and 3, 5 and 6, 5 and 7, 6 and 7, 10 and 11, 14 and 15, 16 and 26, 19 and 20, 19 and 22, 20 and 21, 20 and 22, 21 and 22. After the modification, it was observed that the goodness-of-fit indices of the model provided good validity [26]. The goodness-of-fit indices of the scale items by the confirmatory factor analysis are presented in Table 2. Factor loadings close to each other were assigned to the factors according to the original subscales of the scale. Accordingly, items 1, 2, 3, 4, 5, 6, 7, 8, and 9 were named the “**Attitude**” subscale; items 10, 11, 12, 13, 14, 15, 16, 17, 18, 19, 20, 21 and 22 were named the “**Behavior**” subscale; and items 23, 24, 25 and 26 were named the “**Knowledge**” subscale. Table 2 shows that the goodness-of-fit indices of the scale were significant after modification (*p* = 0.001; *p* < 0.01). The goodness-of-fit indices were NC = 2.451, GFI = 0.852, RMSE = 0.068, CFI = 0.889, NFI = 0.827, RFI = 0.802 and IFI = 0.890. Information on the path diagram and factor loadings of the validated model is presented in Figs. 2 and 3.

### Reliability of the scale

#### Results of the item analysis and internal consistency test

The overall internal consistency coefficient (Cronbach’s alpha) of the Self-Report instrument for Patient Safety Attitudes, Skills, and Knowledge was found to be good (0.875) (Table 3). The item-total correlation values of the items in the scale were more significant than the generally accepted values (expected to be greater than 0.200); however, items 1, 2, 4, 8, and 9 were found to have correlation values lower than the accepted values [22, 23]. However, the internal consistency coefficients achieved by deleting items from the scale separately (Cronbach’s alpha) were not significantly greater than 0.875, the overall internal consistency coefficient. Thus, the analyses were performed without excluding any items or considering future analyses.

**Table 3:** Results of the item analysis

Items	Min–Max	Mean ± SD	Item-Total Correlation	Cronbach Alpha When Item Deleted
**Item 1**	1–5	3.13 ± 1.19	**0.108**	**0.881**
**Item 2**	1–5	3.18 ± 1.11	**0.050**	**0.882**
*Item 3	1–5	2.81 ± 1.12	0.264	0.876
*Item 4	1–5	2.96 ± 1.17	**0.146**	**0.880**
**Item 5**	1–5	3.86 ± 0.79	0.265	0.875
**Item e 6**	1–5	3.92 ± 0.81	0.221	0.876
*Item 7	1–5	3.96 ± 0.77	0.238	0.875
*Item 8	1–5	1.75 ± 0.85	**0.017**	**0.880**
*Item 9	1–5	1.80 ± 0.94	**0.043**	**0.880**
**Item 10**	1–5	3.22 ± 0.89	0.672	0.865
**Item 11**	1–5	3.15 ± 0.92	0.707	0.864
**Item 12**	1–5	2.99 ± 0.99	0.629	0.866
**Item 13**	1–5	3.41 ± 0.91	0.688	0.865
**Item 14**	1–5	3.17 ± 0.99	0.678	0.864
**Item 15**	1–5	3.11 ± 1.03	0.692	0.864
**Item 16**	1–5	2.65 ± 1.16	0.674	0.863
**Item 17**	1–5	3.10 ± 1.04	0.680	0.864
**Item 18**	1–5	3.47 ± 1.02	0.561	0.867
**Item 19**	1–5	3.63 ± 1.04	0.479	0.870
**Item 20**	1–5	3.50 ± 1.04	0.550	0.868
**Item 21**	1–5	2.83 ± 1.20	0.446	0.871
**Item 22**	1–5	3.38 ± 1.11	0.557	0.867
**Item 23**	1–5	3.84 ± 0.74	0.479	0.870
**Item 24**	1–5	3.51 ± 0.94	0.529	0.869
**Item 25**	1–5	3.68 ± 0.83	0.537	0.869
**Item 26**	1–5	3.16 ± 1.10	0.485	0.869

* Items are reverse coded

### Retest reliability of the scale

To determine the time-variability of this methodological study, the scale was applied to 100 nurses at 2-week intervals for the retest reliability of the scale, and the intraclass correlation coefficient (ICC) was calculated (Table 4). The intraclass correlation coefficients were more significant than all items’ generally accepted value (expectedly higher than 0.300) [23]. In general, a strong and statistically significant correlation was found between all the subscale scores and the total score on the scale (*p* < 0.01) (Table 5).

**Table 4:** Retest reliability of the scale

Items	ICC	95% Confidence Interval		F	*p*
		Lower Limit	Upper Limit		
**Item 1**	0.556	0.340	0.701	2.251	< 0.001**
**Item 2**	0.656	0.489	0.768	2.906	< 0.001**
**Item 3**	0.700	0.553	0.798	3.328	< 0.001**
**Item 4**	0.612	0.424	0.739	2.580	< 0.001**
**Item 5**	0.701	0.55	0.798	3.339	< 0.001**
**Item 6**	0.470	0.213	0.644	1.888	< 0.001**
**Item 7**	0.628	0.448	0.750	2.692	< 0.001**
**Item 8**	0.589	0.389	0.723	2.431	< 0.001**
**Item 9**	0.739	0.612	0.824	3.831	< 0.001**
**Item 10**	0.807	0.713	0.870	5.181	< 0.001**
**Item 11**	0.762	0.646	0.840	4.201	< 0.001**
**Item 12**	0.820	0.733	0.879	5.562	< 0.001**
**Item 13**	0.854	0.782	0.901	6826	< 0.001**
**Item 14**	0.701	0.555	0.799	3.340	< 0.001**
**Item 15**	0.834	0.753	0.888	6.025	< 0.001**
**Item 16**	0.710	0.569	0.805	3.447	< 0.001**
**Item 17**	0.810	0.717	0.872	5.258	< 0.001**
**Item 18**	0.805	0.710	0.869	5.117	< 0.001**
**Item 19**	0.855	0.785	0.903	6.903	< 0.001**
**Item 20**	0.764	0.649	0.841	4.237	< 0.001**
**Item 21**	0.703	0.559	0.800	3.369	< 0.001**
**Item 22**	0.819	0.732	0.878	5.536	< 0.001**
**Item 23**	0.727	0.594	0.816	3.662	< 0.001**
**Item 24**	0.734	0.605	0.821	3.764	< 0.001**
**Item 25**	0.740	0.614	0.825	3.849	< 0.001**
**Item 26**	0.818	0.729	0.877	5.483	< 0.001**

*ICC: Intraclass correlation coefficient (ICC)* **p < 0.01

**Table 5:** Correlation evaluation of the subscales and total scales

Dimensions	Attitude	Behavior	Knowledge	Total
	*r*; *p*	*r*; *p*	*r*; *p*	*r*; *p*
**Attitude**	1	-	-	-
**Behavior**	-0.041; 0.463	1	-	-
**Knowledge**	0.021; 0.712	0.545; <0.001**	1	-
**Total**	0.229; <0.001**	0.939; <0.001**	0.705; <0.001**	1

*Pearson correlation analysis* **p < 0.01

## Discussion

Ensuring healthy and sick individuals receive care in a safe environment, protected from neglect and harmful practices, is recognized as a fundamental patient right. In this study, the focus on nurses, who are integral to the healthcare team due to their numerical strength and breadth of service, underscores the critical nature of this research. Adapting the 26-item Self-Report Instrument to Measure Patient Safety Attitudes, Skills, and Knowledge in Turkish has proven valid and reliable, ensuring that it measures these competencies effectively among nurses.

Ensuring validity began with content validity, a crucial step in scale development. In collaboration with experts, the translation and back-translation of the scale assessed its language validity, ensuring that the items are both relevant and representative of the intended features of patient safety competencies. Using the Davis technique, the scale’s Content Validity Index (CVI) was determined to be 0.965, surpassing the accepted threshold of 0.80, indicating that the scale’s items adequately cover the constructs of patient safety attitudes, skills, and knowledge. This finding affirms the scale’s appropriateness for use in the Turkish context and aligns with previous studies emphasizing the importance of a robust content validity process [26–28].

Further, factor analysis was employed to explore the scale’s structural validity, yielding a KMO value 0.965. This result confirms that the scale items are well-correlated and logically grouped, echoing the recommendations for strong factor analysis results [29]. Such validation is essential for applying the scale in varied settings within healthcare, ensuring that the measures reflect true competencies rather than unrelated constructs.

The scale’s reliability was also scrutinized, focusing on internal consistency, foundational for any measurement tool purporting to assess distinct yet related constructs. The overall Cronbach’s alpha coefficient of 0.875 indicates a high level of consistency within the scale, supported by item-total score correlation coefficients. Despite some items showing lower correlation values, the consistency across the scale suggests that it reliably measures the intended patient safety competencies [22–24, 30, 31]. This aligns with findings from Schnall et al., who reported similar internal consistency in the original scale, confirming that our adapted instrument maintains this essential quality [19].

Looking forward, integrating this validated instrument into ongoing training programs for nurses is suggested. Regular use of the scale can facilitate continuous improvement in patient safety practices, addressing current competencies and development areas. Longitudinal studies could further delineate the impact of such educational interventions over time, offering insights into the enduring changes in nurses’ patient safety competencies. This approach not only adheres to the recommended practices in nursing education and patient safety research but also provides a pathway for systematically enhancing the quality of patient care [19, 26, 32].

By embedding these results within the broader context of nursing literature, this discussion underscores the importance of rigorous scale adaptation and validation processes in enhancing patient safety culture. The ongoing application and evaluation of such instruments are imperative in fostering an environment where patient care is continually optimized, reflecting the core values of nursing practice and healthcare delivery.

### Limitations of the Research

The study’s single-center design may not reflect the varied experiences of nurses across different Turkish healthcare settings. The reliance on self-reported data risks response bias, as participants might provide socially desirable answers or inaccurately recall behaviors and attitudes. The two-week interval for test-retest reliability might not capture subtle, long-term changes in attitudes or knowledge, potentially affecting the study’s reliability.

## Conclusions

This study has effectively established the content and construct validity of the Self-Report Instrument to Measure Patient Safety Attitudes, Skills, and Knowledge within a Turkish nursing context, enhancing its applicability for Turkish healthcare professionals. The adaptation of this instrument to Turkish shows promising initial results, suggesting its reliability and validity for assessing nurses’ knowledge and attitudes toward patient safety. However, these findings are preliminary, and ongoing research and further validation in various Turkish healthcare settings are essential.

While the study did not cover the scale’s predictive validity and responsiveness, these represent important areas for future research. Future studies should include longitudinal tracking of patient outcomes and pre-and post-test designs to examine the scale’s responsiveness to interventions. This foundational work is crucial for enhancing patient satisfaction and quality of life by improving patient safety culture.

## Data Availability

Yes, I have research data to declare.
